# “Complex” Vasovagal Syncope: A Zebra Among Horses

**DOI:** 10.3389/fneur.2020.550982

**Published:** 2020-12-16

**Authors:** Anwer Zohaib Siddiqi, Derrick Blackmore, Zaeem Azfer Siddiqi

**Affiliations:** Division of Neurology, Department of Medicine, University of Alberta, Edmonton, AB, Canada

**Keywords:** syncope, seizures, asystole, bradycardia, vasovagal syncope

## Abstract

**Background:** Vasovagal syncope (VVS) occurs due to cerebral hypoperfusion from a fall in blood pressure, with accompanying bradycardia in most cases. Seizure and/or asystole may accompany VVS, though their prediction within the VVS cohort remains elusive.

**Objective:** To further characterize VVS and to find predictive features of “complex” VVS (defined as VVS associated with seizures and/or asystole).

**Methods:** We reviewed medical records of all patients who were referred for orthostatic intolerance and had a definite VVS during the head-up tilt table testing (HUTT). The following variables were recorded: cardiovascular indices during HUTT, autonomic testing results, and semiology of asystole and/or seizure when present. Simple frequency and correlation analysis were performed using the ANOVA.

**Results:** A total of 78 independent VVS were recorded in 60 patients of which 24% were not preceded by presyncope. Vasodepressor (45%) and mixed (38%) VVS were the most prevalent types. Eighteen (23%) were complex VVS; five had an associated seizure (SySz), nine were accompanied by asystole (SyAs), and four had both (SySzAs). Males were significantly more likely to have complex VVS. Mean asystole duration was somewhat longer in the SyAsSz group. The severity of bradycardia significantly correlated with complex VVS and was a predictor of SySz. Autonomic abnormalities were frequent but did not distinguish the two VVS subgroups. Seizures had multiple distinguishing features from those typically associated with epileptic seizures.

**Conclusions:** The underlying pathophysiologic mechanisms of complex VVS remain unclear, but the severity of cerebral hypoperfusion due to bradycardia likely plays a key role in seizure generation.

Vasovagal syncope (VVS) may be described as a “transient loss of consciousness and postural tone resulting from global cerebral hypoperfusion with spontaneous and complete recovery and no neurological sequelae” (1–3). The estimates of syncope range from 18.1 to 39.7 episodes per 1,000 patients per year, but this incidence significantly increases after the age of 70 years (4). Syncope can have serious adverse effects on quality of life by reducing mobility and usual activities and by increasing depression, pain, and risk of physical injury. By doing so, it can significantly increase direct and indirect social healthcare costs. While VVS may, at times, be homeostatically protective (5), the most prevalent form is debilitating syncope (2–4) and results from abnormal cardiovascular reflexes that cause vasodilation, bradycardia, and a resulting fall in systolic blood pressure and hypoperfusion of the brain. While reflex syncope may be caused by dysfunction in either parasympathetic or sympathetic efferent pathways, autonomic efferents remain largely intact (6). It is common for patients that undergo VVS to have “pre-syncopal” phase or prodrome, which can include nausea, light headedness, blurry vision, or pallor.

Providing orthostatic challenge can be a useful technique for diagnosing VVS. Normally, during standing 300–800 mL of blood pools in the lower limbs, inducing baroreceptor reflexes, encouraging sympathetic activation and maintenance of blood pressure (4). Head-up tilt table (HUTT) testing, first described by Allen et al. in 1945 (7), is the gold standard for diagnosing syncope. By tilting a patient between 60 and 90°, orthostatic stress and sympathetic activity are maximal (8). HUTT can differentiate between symptomatic and asymptomatic patients (9), reflex syncope, orthostatic hypotension, and pseudosyncope and has been estimated to have a sensitivity of 65% and a specificity of 92% (8, 10). The HUTT is also useful in distinguishing different types of VVS.

The co-occurrence of VVS and seizures and/or cardiac asystole, that is, “complex VVS,” is well-known though the underlying pathophysiology remains unclear. Up to 91% of individuals with syncope may develop a rigid posture and have some myoclonic jerking activity while about 6–25% of individuals with vasodepressive syncope may have convulsive episodes (11, 12). In a retrospective review in 226 individuals with syncope during HUTT, 5.8% of patients had seizures (13). Another feature of VVS is cardiac asystole, which has a variable incidence, ranging from 9.1 to 24% (12, 14). The pathophysiological mechanisms underpinning both pharmacologically provoked and spontaneous VVS/VVS-asystole in otherwise healthy individuals remain unclear. Females tend to have a longer duration of asystole, which could be predicted by heart rate variability (a measure of cardiac autonomic tone) (15).

We hypothesized that as compared to patients simple VVS, those with complex VVS, may demonstrate abnormalities on dedicated autonomic testing, that is, heart-rate variability, sympathetic dysfunction, cardiac arrhythmias around the event, etc., that may not be necessarily causative but associative and may provide further insight into the variable presentation of the two types of VVS. Hence, the objective of the present retrospective study was to characterize features of complex VVS occurring during routine head up tilt table test (HUTT) in otherwise healthy patients. Specifically, we sought to ascertain distinguishing features of VVS with or without seizures and/or asystole and identify any demographic or autonomic factors predictive for simple/complex syncope.

## Methods

### Study Participants

Medical records of consecutive patients between 2009 and 2016 who were referred to University of Alberta Hospital autonomic laboratory for assessment of orthostatic intolerance and/or syncopal episodes were reviewed. Patients who suffered a definite VVS (See Methods/Definitions c.1) during the HUTT were included in this study. These patients were referred by their neurologist, cardiologist, or primary care physician. All patients had a normal ECG, and several have had extensive cardiac workup, including transthoracic echocardiogram and Holter monitoring, and EEG prior to be assessed at the autonomic laboratory. The project was approved by the Human Research Ethics Board, University of Alberta (Application number Pro00073273).

### Laboratory Testing

We used the Mayo Clinic battery for autonomic testing (see below), which includes the Quantitative Sudomotor Axon Reflex Test (QSART), cardiovagal tests, that is, heart-rate response to deep breathing (HRDB) and Valsalva Maneuver (VM), and the HUTT test. All patients were asked to avoid stimulants for 24 h prior to testing.

#### HUTT (16)

The HUTT was conducted in a quiet, dimly lit room, and patients lay supine for at least 30 min prior to the HUTT. Arterial blood pressure was measured continuously with a finger plethysmograph (Nexfin HD, BMEYE, Amsterdam, The Netherlands) and manually from the opposite arm. Heart rate was recorded continuously with a three-lead ECG. After baseline recording for 2–5 min, patients were tilted to an angle of 70° for 60 min, after which patients were reclined back to supine position for another 5 min. The brachial blood pressures were recorded at 30 s, 1 min, 2 min, and then at 5-min intervals till the end of the test. Symptoms and signs of presyncope were monitored and recorded throughout the duration of HUTT.

##### Quantitative sudomotor axon reflex testing (QSART)

Postganglionic sympathetic sudomotor axon integrity was evaluated through QSART (17). Sweat responses were recorded from four consistent sites (left forearm, proximal lateral leg, medial distal leg, and proximal foot) using the Q-Sweat sudorometer (Quantitative Sweat Measurement System; WR Medical Electronics Co., Stillwater MN, USA) following the preparation and acquisition protocols as described by Sletten et al. (18). Test interpretation involved comparison of observed sweat volumes (in nl/min) with age- and gender-based norms.

##### Cardiovagal (parasympathetic) testing

Parasympathetic (cardiovagal) function was assessed by measuring HRDB, VM, and HUTT. The responses were compared to the age-based norm (19, 20). Briefly, for the HRDB test patients the patients were required to inhale deeply and evenly and then exhale fully, each phase was performed over 5 s and was paced to an oscillating LED, completing one cycle in 10 s. The patients would complete a total of eight inhalation/exhalation cycles. Further, the first set of eight deep breaths was followed by a rest period of 2 min, and the whole set of eight deep breaths was repeated. Beat-to-beat R-R intervals were recorded during each effort and the mean HRDB range was determined by averaging the largest R-R differences for five consecutive cycles. During the VM, patients blew into a tube at a constant pressure of 40 mmHg for 15 s while supine. The Valsalva ratio (VR) calculated by dividing the maximum heart rate (HR) generated by lowest HR occurring within 40 s after VM. The maneuver was performed twice, and the best response recorded. Finally, during HUTT the 30:15 ratio was calculated by dividing R-R interval at 30 s to the RR interval at 15 s while the patients were inclined.

### Definitions

#### VVS

The VVS was defined as loss of consciousness concurrently with a drop in systolic blood pressure of ≥ 30 mm of Hg to differentiate from psychogenic syncope. Asystole was defined as an R-R interval ≥ 3 s, as in previous studies (12, 20). The asystole was also confirmed on the arterial blood pressure recording through the finger plethysmograph, which showed absence of arterial pulse wave. The duration of asystole was recorded until normal sinus rhythm and the arterial pulse wave was restored. VVS associated with either a seizure and/or asystole was termed as “*complex syncope*” as compared to a “*simple syncope*,” which was accompanied by neither. Patients who had “near” syncope (vasodepressor response without loss of consciousness) were excluded.

#### Presyncope

The onset of the presyncopal phase was defined as the time point preceding the actual VVS when any of the following symptoms or signs first appeared: feeling of about to lose consciousness, light-headedness or dizziness, vertigo, weakness, diaphoresis, epigastric discomfort, nausea, blurred or faded vision, pallor, or paresthesias. Time interval from the start of HUTT to presyncope and to syncope were recorded.

#### Types of Syncope

VVS was subcategorized based on the associated blood pressure and heart rate changes (20).

- *Mixed syncope (Type I)* was defined as significant drop in blood pressure before a mild drop in heart rate (not below 40 beats/min for longer than 10 s).
- *Cardioinhibitory syncope (Type II*) was marked by a drop in heart rate below 40 beats per minute for longer than 10 s and/or asystole ≥3 s. In *Type IIA*, the blood pressure drop occurred before the drop in heart rate; in *Type IIB*, blood pressure and heart rate dropped simultaneously.
- *Vasodepressor syncope (Type III)* was characterized by pure, significant hypotension accompanied by slight bradycardia.

### Convulsive Syncope

Any myoclonic, tonic, or clonic activity accompanying the VVS was documented by the technologist performing the HUTT and semiology of the ictal and post-ictal phase was recorded.

### Postural Orthostatic Tachycardia Syndrome (POTS)

Postural Orthostatic Tachycardia Syndrome (POTS) was defined as an excessive increase in heart rate (>30 beats per minute from baseline and/or heart >120/min) within 10 min of assuming an upright posture (21) without any orthostatic hypotension (defined as drop in systolic blood pressure by >20 mm of Hg).

### Statistical Analysis

Statistical analysis was performed using IBM SPSS Statistics 23.0 software (SPSS, Chicago, IL, USA). For all statistical tests, significance was determined at *P* < 0.05. Scatterplots and contingency tables were used initially to explore possible relationships. Promising factors were then analyzed to further confirm relationships between variables of interest. Two-tailed independent-samples *t*-tests were used to compare continuous variables, the chi-square test for independence to compare categorical variables and logistic regression to compare relationships between continuous and categorical variables and calculate odds ratios.

## Results

The demographic data of our patients is presented in Table 1. A total of 78 VVS episodes were recorded during HUTT in 60 patients (mean age: 46 years). Some patients had more than one HUTT during the study period. No patients developed orthostatic hypotension or cardiac arrhythmia except during recovery from asystole where some patients had a few ventricular beats appear before resumption of normal sinus rhythm. Of the total VVS episodes, 60 (77%) were simple, while 18 (23%) were complex VVS being associated with either a seizure (SySz), asystole (SyAs), or both (SySzAs). Males were significantly more likely to have complex syncope (*p* < 0.03). The mean time to VVS from the start of HUTT was about 25 min (range 10–40 min) though it occurred earlier in patients who had a seizure with or without asystole (mean: 20 min). Overall, the mean blood pressure reduced by 50% and the heart rate reduced by 38% from the baseline during the VVS. In 10 patients, the findings on HUTT were consistent with POTS, all had simple syncope. Vasodepressor (45%) or mixed (38%) syncope were the most prevalent types in our cohort followed by the cardioinhibitory type (17%). The vasodepressor and mixed syncopal episodes occurred mainly in the simple group. Age of the patients did not correlate with the type of VVS though males were significantly more prone to have complex VVS.

**Table 1:** Demographic Data and Syncope Characteristics for patients with simple and complex syncope.

	**All**	**Simple VVS**	**Complex VVS**		
			**Asystole**	**Seizure**	**Asystole + seizure**
Number of participants	60	42	9	5	4
Mean age (years)	46.0 ± 22.0	49.0 ± 23.4	46.2± 17.2	37.3 ± 16.9	30.8 ± 16.9
Men/women	21/39	13/29	5/4	3/2	0/4
Number of VVS	78	60 (77%)	9 (12 %)	5 (6%)	4 (5%)
% SBP drop from baseline	52.1 ± 15.4	51.6 ± 14.9	55.3 ± 17.4	53.6 ± 24.7	34.7 ± 14.6
% HR drop from baseline	38.4 ± 25.0	33.7 ± 23.2	57.3 ± 20.4	39.0 ± 26.9	65.8 ± 28.2
Mean time to VVS (min.)	24.9 ± 15.5	25.0 ± 16.2	30.3 ± 16.6	18.4 ± 7.86	21.1 ± 8.73
Mean presyncope time (min.)	8.03 ± 9.60	8.99 ± 10.1	3.85 ± 3.64	3.7 ± 1.64	11.3 ± 17.5
Mean asystole duration (sec.)	N/A	N/A	10.6 ± 5.81	N/A	13.9 ± 11.2
# of VVS without Presyncope	19 (24%)	17	1	0	1
Type of VVS^*^					
Vasodepressor	34 (45%)	32	0	2	0
Mixed	30 (38%)	25	2	3	0
Cardioinhibitory type A	4 (5%)	1	3	0	0
Cardioinhibitory type B	9 (12%)	2	4	0	3

* *One HUTT had artifact which precluded classification*.

### Presyncopal Phase

Presyncopal symptoms occurred in 59 cases at about 8 min (range: several seconds to 18 min) into the HUTT. In 19/78 (24%), the syncope occurred abruptly without any preceding symptoms/signs of presyncope. Most of these (85%) were followed by a simple syncope. Presyncopal symptoms tended to occur earlier in the complex groups (SyAs: 3.85 ± 3.64 min; SySz: 3.7 ± 1.64 min) compared with the simple syncope (8.99 ± 10.3) cohort, though this difference was not statistically significant.

### Simple/Complex Syncope

All patients had normal sinus rhythm at baseline, and no cardiac arrhythmia was noted throughout the HUTT. Asystole occurred in 13/78 (16%); four (6%) experienced a seizure concurrently with the syncope. Sinus rhythm was restored spontaneously in all cases, with some having few ventricular beats before restoration. The mean asystole duration did not change significantly if it was accompanied by a seizure, that is, SyAsSz. The relative reduction in systolic blood pressure from baseline (about 55%) during the vasodepressor phase was similar in both simple and complex syncope groups except in the SySzAs group (i.e., 35%), likely due to muscle contractions. As expected, overall reduction in heart rate from baseline was much higher in the complex group (p < 0.001).

### Predictors of Simple/Complex Syncope

Using logistic regression, the likelihood of complex syncope was predicted by being male (OR 3.615, 95% CI 1.192–10.964; *p* = 0.023) and abnormal VRs. While significant, percent decrease in heart rate at syncope (OR 0.959, 95%, CI 0.933–0.987; *p* = 0.004) and R-R interval duration (OR 0.998, 95% CI 0.998–0.999; *p* = 0.0003) had very little effect on the likelihood of either simple or complex syncope.

### Seizure

Overall 9/78 (12%) had convulsive syncope with most (85%) preceded by presyncope. Three episodes were associated with urinary incontinence. No complex automatisms (chewing movements, lip smacking, grasping, or fumbling with hands), tongue biting or foaming from the mouth were documented. Most had a short (< 5 min) period of post-ictal confusion or disorientation. Only one patient had somewhat prolonged post-ictal phase characterized by fatigue, confusion, and sleepiness lasting several minutes.

### Predictors of Seizure

Overall, demographics or sympathetic and parasympathetic parameters on autonomic testing were poor predictors of syncopal seizure. Only increases in R-R duration were statistically significant for changes in the risk of syncopal seizure, albeit not clinically (OR 1.000, 95% CI 0.999–1.000; *p* = 0.050).

### Autonomic Dysfunction

Results of comprehensive autonomic testing are shown in Tables 2, 3. Autonomic testing was available in 73 cases. Of these 28 (38.4%) had abnormal sweat responses, more prevalent in the complex VVS group (50.0% vs. 34.5%; *p* = 0.159). The HRDB was abnormal in 13/73 (17.8%), similar in the simple and complex groups (18.2% vs. 16.7%; *p* = 1.00). No members of the complex VVS group showed any abnormality on the VM as compared to 20% in the simple group that did (*p* = 0.051).

**Table 2:** Results of autonomic testing in all patients.

**Type of syncope**	Autonomic laboratory testing^*^									
	**QSART**		**HRDB**		**Valsalva**		**HUTT (30:15)**	
	**Normal**	**Abnormal**	**Normal**	**Abnormal**	**Normal**	**Abnormal**	**Normal**	**Abnormal**
Total	45 (61.6%)	28 (38.4%)	60 (82.2%)	13 (17.8%)	63 (85.1%)	11 (14.9%)	43 (58.1%)	31 (41.9%)
Simple	36 (65.5%)	19 (34.5%)	45 (81.8%)	10 (18.2%)	45 (80.4%)	11 (19.6%)	33 (58.9%)	23 (41.1%)
Complex	9 (50%)	9 (50%)	15 (83.3%)	3 (16.7%)	18 (100%)	0 (0%)	10 (55.6%)	8 (44.4%)

* *Nine patients had Postural Orthostatic Tachycardia Syndrome, all had simple syncope*.

**Table 3:** Chi-Square Comparison of Autonomic Testing for Each Syncope Group.

	**Simple syncope**	**Complex syncope**	**Sig. (2-tailed)**
QSART	19	9	0.159
HRDB	3	10	1.00
Valsalva	0	11	0.051
30:15	8	22	0.558

### Predictors of Asystole

Predictors of asystole were statistically significant, but not clinically. The percent HR decrease at syncope (OR 1.059, 95% CI 1.020–1.100; *p* = 0.002) and increased R-R interval duration (OR 1.000, 95% CI 1.001–1.002; *p* = 0.005) were associated with marginal increases in the risk of asystole at the time of syncope. The associations between the predictor variables and outcomes are shown in Table 4. The quoted *p*-value is that for the stepwise predictor variable for the given logistic regression model.

**Table 4:** Correlations Between VVS, Demographic and Autonomic Laboratory Testing.

	**Simple/complex**			**Seizure**			**Asystole**		
	**Sig**.	**OR**	**95% C.I**.	**Sig**.	**OR**	**95% C.I**.	**Sig**.	**OR**	**95% C.I**.
**DEMOGRAPHICS**									
Age	0.501	1.008	0.983–1.035	0.194	0.973	0.935–1.013	0.742	0.995	0.966–1.024
Gender	*0.023*	3.615	1.192–10.964	0.720	0.760	0.172–3.349	0.123	0.380	0.111–1.300
**TILT TEST MEASURES**									
Duration of presyncope	0.821	1.000	0.999–1.001	0.578	0.999	0.998–1.001	0.622	1.000	0.999–1.001
Post-tilt time to syncope	0.992	1.000	0.999–1.000	0.273	0.999	0.998–1.000	0.525	1.000	0.999–1.000
% BP decrease at syncope	0.811	1.004	0.969–1.04	0.291	0.973	0.925–1.023	0.660	0.990	0.951–1.031
% HR decrease at syncope	*0.004*	0.959	0.933–0.987	0.104	1.025	0.994–1.058	*0.002*	1.059	1.020–1.100
R-R interval duration	*0.003*	0.998	0.998–0.999	*0.050*	1.000	0.999–1.000	*0.005*	1.001	1.000–1.002
**AUTONOMIC LABORATORY TESTS**									
QSART	0.245	0.527	0.179–1.551	0.265	2.228	0.543–9.130	0.993	1.005	0.293–3.448
HRDB	0.884	1.111	0.269–4.58	0.712	1.376	0.251–7.538	0.801	0.809	0.156–4.185
Valsalva	*0.040*	0.110	−2.775–2.989	0.180	4.010	1.095–6.923	0.097	6.150	3.254–9.042
30:15	0.801	0.871	0.298–2.543	0.121	3.199	0.733–13.962	0.375	0.560	0.155–2.016

*Bold, italicized, underlined values represent statistically significant values (p ≤ 0.50)*.

## Discussion

To date, understanding of the complex factors associated with syncope remains incomplete (16). The objective of this study was to further characterize complex VVS patients with orthostatic intolerance. Our results indicate seizures, a brief period of asystole or both accompany a sizeable number (20%) of syncopal episodes, predominantly in males. All asystole episodes resolved spontaneously, and the severity of bradycardia during VVS strongly correlated with occurrence of seizure. Vasodepressor and mixed syncope types were the most prevalent types of VVS. In about 25% of patients, VVS occurred abruptly without any preceding warning symptoms and signs. There was a high prevalence of autonomic abnormalities in patients with VVS, though this did not appear to increase the predisposition to asystole or seizures.

Previous reports have shown a highly variable incidence of asystole during VVS on HUTT, which is likely due a number of factors, including the duration used to define asystole, duration of the HUTT, angle of tilt, or whether any pharmacologic agent was used during HUTT for increasing the sensitivity of the test. In our study, about 20% of patients with VVS experienced an asystole lasting ≥ 3 s (including those with and without seizure). Other studies have used ≥ 5 s duration as the definition of asystole, though it may be noted that only two in our cohort had asystole lasting <5 s. One study in which patients were tilted at 80° for 30 min, of 242 patients with a positive HUTT (pre-syncope and/or syncope) only three had prolonged asystole, all of whom had cardioinhibitory syncope (22). The definition used for asystole was unclear. In another study, 13% (10/77) of patients experienced asystole (defined as an RR interval ≥ 5 s) when tilted to 60° for 60 min (23) Other studies have quoted an incidence of 9 to 12% of patients having an asystolic response (RR interval ≥ 5 s) (12, 24). Experiencing asystole during HUTT is also subject to day-to-day variability, which makes it difficult to ascertain the true rate among individuals with VVS (12). The mean asystole duration in our cohort, with or without seizure, was similar to that reported previously for asystole followed by seizure (12.8 s) (23). Some studies have shown that the average age (range 21–47 years) for syncope without asystole is higher than syncope with asystole (15–32 years) (23–26). Our data does not corroborate this relationship of complex VVS with age, though male gender increases the predisposition to complex syncope, that is, SySz.

About 20–30% of patients diagnosed with epilepsy on clinical grounds have non-epileptic convulsive disorders, many having cardiac syncope as the underlying cause (27, 28). The frequency of VVS being misdiagnosed as epilepsy has been reported to be 13% in patients referred to a large tertiary epilepsy clinic (29). Others have reported incidence of about 5–6% (13, 14). Many patients receive antiepileptic medications for several years before being correctly diagnosed (30). In our population, 13.6% of individuals (with and without asystole) experienced seizure-like activity during VVS. Our data and others indicate that HUTT may be quite useful in discerning patients suspected to have non-epileptic convulsions (31). Typical prodromal symptoms usually precede most VVS associated with seizures. Other clinical features that favor syncopal over epileptic seizures include occurrence during upright posture (i.e., during the HUTT), lack of complex automatisms (21). shorter duration of the convulsive phase, lack of tongue biting, and short duration (few minutes) of post-ictal confusion. It may be noted that urinary incontinence maybe associated with seizures in the setting of VVS and does not differentiate an epileptic seizure.

Why some patients have seizures during VVS is unclear, and the predictive factors remain unidentified. Concurrent EEG with HUTT have shown a change in EEG activity during vasodepressor and cardioinhibitory responses during HUTT (32–34). In our cohort, a notable feature was that the cardioinhibitory response was significantly severe in the VVS accompanied by seizure. Hence, the severity of bradycardia, as shown by the length of R-R interval, was a key predictor of seizures. Furthermore, of the nine patients who had seizures 75% (6/8, one could not be subclassified due to artifact) had either cardioinhibitory or mixed type of VVS rather than the more common vasodepressor type. These observations suggest that the severity of cardioinhibitory response with associated cerebral hypoperfusion from bradycardia may be the determining factor in generation of seizures during VVS.

Prior studies have reported that VVS typically occurs in subjects who have otherwise normal autonomic responses (35). Others have suggested an important role of autonomic nervous system based on variable pattern of autonomic responses using spectral analysis of HRDB detected in patients with VVS as compared to healthy controls (36). Our cohort showed patchy autonomic abnormalities of the sympathetic and parasympathetic responses in a sizeable number of VVS patients who were otherwise healthy. None of our patients demonstrated any significant orthostatic hypotension on HUTT or markedly reduced HRDB. It is plausible that the impaired peripheral autonomic reflexes lead to gradual pooling of the blood in the lower body with maintenance of upright posture leading to reduced venous return and cardiac output. Further studies are needed to assess the contribution and role of these autonomic abnormalities in VVS.

The characterization of VVS into mixed, vasodepressor, and cardioinhibitory syncope is of practical value. Although the pharmacological management of both vasodepressor and cardioinhibitory VVS is relatively similar, cardiac pacing maybe beneficial for intractable cardioinhibitory syncope (22). Unfortunately, there is little agreement in the relative proportion of each syncope type in patients with VVS among various studies. In a study on 34 patients with syncope, 13 had cardioinhibitory, 10 had vasodepressor, and 11 were afflicted with mixed syncope (37). Others have found higher proportion of cardioinhibitory type (40.7%) and vasodepressor types (59.3%) of syncope (11). In the present study, vasodepressor and mixed syncopes were the most common, which is in accordance with some previous reports (25, 38) but discordant with others that show that the rate between the three types of syncope is relatively equal. The apparent variability of VVS types likely reflects the population studied, age of the patients, and possibly, the HUTT procedures used. Larger multicenter studies with uniform protocols are needed to resolve the factors, which underlie this discordance.

In summary, our results indicate complex VVS is relatively common and that seizures may be related to the severity of cardioinhibitory phase that accompanies VVS. The relatively small sample size precludes definitive conclusions, and larger studies are needed to confirm these novel findings. Employment of complementary techniques such as EEG and spectral analysis of HRV may provide more insight into the underlying pathophysiology of complex VVS.

## Data Availability Statement

The original contributions presented in the study are included in the article/supplementary materials, further inquiries can be directed to the corresponding author/s.

## Ethics Statement

The studies involving human participants were reviewed and approved by Health Research Ethics Board, University of Alberta. Written informed consent for participation was not required for this study in accordance with the national legislation and the institutional requirements.

## Author Contributions

AS collected the data, helped in analysis, and wrote the manuscript. DB performed the testing and helped in data analysis edited the manuscript. ZS supervised the project, analyzed the data, and edited the manuscript. All authors contributed to the article and approved the submitted version.

## Conflict of Interest

The authors declare that the research was conducted in the absence of any commercial or financial relationships that could be construed as a potential conflict of interest.
